# Breaking with the Criteria; Selective Mutism and its Forbidden Connection with Autism

**DOI:** 10.1007/s10802-025-01414-x

**Published:** 2026-01-09

**Authors:** Ina Helgesen, Anders Nordahl-Hansen

**Affiliations:** 1 Department of Education, ICT and Learning, Østfold University College, Postboks 700, NO-1757 Halden, Norway; 2 Department of Educational Science, University of South-Eastern, Vestfold, Norway

**Keywords:** Selective mutism, Autism, Co-occurrence, ICD-11, Diagnosis, Gender distribution

## Abstract

Despite autism being defined as an exclusion criterion for selective mutism (SM) in the European diagnostic manual, many studies have revealed a significant overlap between these conditions (Keville et al., 2023; Muris & Ollendick, 2021; Sharkey & McNicholas, 2012; Suzuki et al., 2020). The purpose of this study was to examine selective mutism in Norway using data from the Norwegian Patient Register (NPR), with a specific focus on quantifying its co-occurrence with Autism Spectrum Disorder (ASD). We have identified a sample (*n* = 1,682), aged from 3 years to 18 years in Norway, who during the period from January 1, 2008, to April 30, 2023, have had at least one documented episode where the diagnosis of selective mutism was registered. Many individuals show a clear overlap between selective mutism and autism, at 11.7%. The Norwegian gender ratio in this SM group was 2.13 girls for every boy (M/F 1:2.13). The exclusion of autism as a co-occurring diagnosis with selective mutism in ICD-10/11 may lead to delayed or incorrect diagnoses, preventing early intervention and tailored support. This particularly affects children who experience both conditions but initially present with SM as the dominant clinical feature.

## Introduction

Selective mutism (SM) is an anxiety disorder characterized by a persistent failure to speak in specific social situations despite adequate speech elsewhere. Emphasis is placed on duration, indicating that it is not just at the start of school (World Health Organization (WHO), 2024). Both the current ICD-11 (WHO, 2024) and its predecessor ICD-10 (WHO, 1992) stipulate that SM should not be diagnosed if the speech difficulties are better explained by autism spectrum disorder (ASD).

Despite autism being defined as an exclusion criterion for SM, studies such as Steffenburg et al. (2018) have revealed a significant overlap. Similar findings have been reported (Keville et al., 2023; Muris & Ollendick, 2021; Sharkey & McNicholas, 2012; Suzuki et al., 2020). The diagnostic manuals DSM-5 and ICD-11 have different approaches to the relationship between autism and SM, creating a challenge in the diagnostic process.

### The Diversity in Selective Mutism

The term ‘mutism’ refers to the absence of verbal communication without any apparent damage to the speech area. In this context, ‘selective’ indicates that the inability to speak occurs only in specific social settings, such as at school or directed towards certain individuals (Rozenek et al., 2020). This situational mutism can be exemplified by a child with SM talking with a parent in town and suddenly becoming completely silent mid-sentence when the child with SM observes someone from school approaching.

Affected individuals gradually withdraw linguistically from their surroundings. This gradual withdrawal from social interaction is unique to selective mutism and distinguishes it from other anxiety disorders (Rogoll et al., 2018). The beginning of mutism during early childhood can affect development and have a bad influence on school performance. It’s important for healthcare professionals and educators to know about SM, because starting early intervention leads to better outcomes (Rozenek et al., 2020).

Kearney and Rede (2021) argue for a more nuanced approach to selective mutism based on its diversity and complexity. They argue that SM, despite being classified as an anxiety disorder, can be better understood as a neurodevelopmental disorder. This is supported by observations of central nervous system development and epigenetic foundations, as well as atypical neurodevelopment associated with communication disorders. Kearney and Rede (2021) also highlight SM as a potential crucial turning point in development, which can impact other areas of a child’s functioning and intensify the disorder. Their conclusion suggests the need for an expanded approach, both in clinical and school-based settings, by considering the child’s overall development (Kearney & Rede, 2021).

Cohan et al. (2008) examined the need for a more precise classification of selective mutism, given its considerable variation. They argue for the use of clinical subgroups to better understand SM and tailor intervention to individual needs, citing successful use of such specifications for other conditions, exemplified by depression and social anxiety. Their study included 130 children with SM aged 5 to 12 years, and they proposed three main groups after analysis: (1) an exclusively anxious group where social anxiety was the most prominent feature, (2) an anxious-oppositional group where both anxiety and low-level behavioral problems were prominent, and (3) an anxious-communication-delayed group where both anxiety and developmental language delays were prominent. The results indicated that children in the anxious-communication-delayed group had higher levels of selective mutism symptom severity and externalizing problems compared to children in the exclusively anxious group.

Another study by Stein et al. (2011) found that the gene rs2710102 in CNTNAP2, associated with the neurexin superfamily, is linked to increased risk of selective mutism and social anxiety-related traits. The gene consistently suggests association with SM in families and social anxiety in young adults. The study suggests a partially shared genetic basis between autism, selective mutism, and social anxiety disorder. Stein et al. (2011) urged caution in reclassifying SM under anxiety disorders in DSM-5, as diversity in SM may link some forms closer to the autism spectrum.

Steinhausen and Juzi (1996) discovered that 38% of children with SM had some form of speech or language disorder, with expressive language disorders (28%) and articulation disorders (20%) being the most common types. Kristensen (2000) examined the cooccurrence associated with SM in children, revealing significant overlaps with developmental disorders (68.5%) and anxiety disorders 74.1%. A notable finding was that 46.3% of children with SM had both an anxiety diagnosis and a developmental disorder/delay. Driessen et al. (2020) found that 80% of children with SM had at least one concurrent anxiety disorder, with social phobia being the most prevalent (69%). Others included specific phobias (19%), separation anxiety (18%), generalized anxiety disorder (6%), and obsessive-compulsive disorder (OCD) (6%). There are few studies available on selective mutism and ADHD, but as Surén et al. (2012) note that the overlap between autism and ADHD is also a discussed topic. DSM-IV and ICD-10 exclude concurrent diagnoses of these disorders, but many studies have found extensive clinical overlap. Between 20% and 50% of children with ADHD meet the criteria for autism, and 30% to 80% of children with autism meet the criteria for ADHD (Surén et al., 2012).

### Selective Mutism Co-Occurring with Autism

According to ICD-11 autism is a neurodevelopmental condition characterized by persistent difficulties in social interaction and communication, alongside restricted and repetitive behaviors. Traits typically emerge in early childhood but may become more apparent as social demands increase (Davis et al., 2022). The condition significantly affects daily functioning across personal, social, and educational domains, though its presentation varies among individuals. Autism encompasses a wide range of intellectual and language abilities (ICD-11; World Health Organization, 2024).

The autistic experience of shutdown can in some cases be mistaken for selective mutism. Vogel et al. (2022) showed that children with SM display a socially triggered fear response, during an imposed speaking task. They froze and made 20% fewer facial fixations than controls, indicating gaze avoidance rather than sensory overload. By contrast, Belek (2018, pp. 35–36) reports that autistic adults often experience shutdown as complete loss of speech and movement, when exposed to sudden noise, harsh light or unexpected touch.

Phung et al. (2021) examined shutdown in youth with autism, where they used analogies to describe experiences of feeling “exhausted and/or frozen”. Several participants identified loud classroom environments as a primary shutdown trigger (Phung et al., 2021). These studies indicate that SM “freeze” is primarily socially triggered, whereas autistic shutdown is often driven by sensory overload.

In a study on autistic masking, it is shown how the narrative of stigma and the illusion of choice play a role (Pearson & Rose, 2021). Although masking is described as a “social strategy”, it is important to acknowledge how social environments and norms affect autistic people. Masking is not only visible tactics like eye contact and mimicry of facial expressions but also a gradual development influenced by external pressures. They argue that research must consider the development of masking over time. Furthermore, this approach highlights how autistic masking is not just a personal experience but also a response to the broader social environment. Autistic individuals develop masking techniques not only to navigate social situations, but also as a reaction to the stigmas and expectations imposed on them by society (Pearson & Rose, 2021).

In cases of overlap between SM and autism, this can be challenging to detect in a school context if silence is very prominent. Like masking, the silence in SM may conceal the autistic characteristics at school, but they may become more evident in the home environment. Such as the study conducted by Rødgaard et al. (2021a), which examined the prevalence of childhood diagnoses among individuals diagnosed with autism in adulthood, can shed light on this masking. They used data from the Danish National Patient Register to identify individuals diagnosed with autism as adults, as well as a control group without autism diagnosis. The aim was to investigate whether a late autism diagnosis could be explained by misdiagnosis in childhood or diagnostic overshadowing. The results showed that many childhood diagnoses were overrepresented among those with an adult autism diagnosis. ADHD, affective disorders, anxiety, and stress disorders were the most prevalent in this group. However, 69% of men and 61% of women with an adult autism diagnosis had not received any of the investigated diagnoses before the age of 18, and most childhood diagnoses were given after the age of 12. The study also showed that cases of ADHD, mood disorders, and anxiety were frequent before the age of 18 (Rødgaard et al., 2021a).

Early case work already hinted at an overlap between SM and autism. In a Swedish study, one of two personally examined SM cases met almost all Asperger criteria (Kopp & Gillberg, 1997). The authors proposed that SM might (a) cluster in families with autism or (b) represent a milder variant of autism. DSM-IV states that selective mutism should not be diagnosed if the disturbance occurs exclusively within a pervasive developmental disorder. However, they found that this criterion was difficult to apply, as SM is not typically a feature of Asperger’s syndrome. If there is a genuine relationship between selective mutism and Asperger’s syndrome, it cannot be confirmed if both conditions cannot be diagnosed in the same individual (Kopp & Gillberg, 1997).

Clinic‑ and population‑based studies since 2001 reinforce a possible link. In a Norwegian family study (*N* = 54) 74% of children with SM carried another anxiety disorder and 7% met Asperger criteria (Kristensen & Torgersen, 2001). A chart review from Gothenburg (*N* = 97, 4–18 year) found that 63% of children referred for SM satisfied full autism criteria when re‑evaluated with multidisciplinary assessment (Steffenburg et al., 2018). Average diagnostic age was three years for SM‑only and almost five years for SM + ASD, despite symptom onset between ages 2 and 5. However, the onset of SM symptoms in those with concurrent autism often starts significantly later, on average 18 months later. This may be explained by SM either “caused by” autism or overshadowed by other autism traits. Despite early symptom onset, early diagnosis is not achieved. The average age for SM diagnosis was approximately 3 years after symptom onset for those with SM alone, and almost 5 years later for those with both SM and autism. It is also relevant to note that the age range for SM diagnosis varied from 4 to a18 years, and for some children, more than 4 years could pass between symptom onset and diagnosis (Steffenburg et al., 2018).

Co-occurrence further complicates the picture. Case studies report SM co‑occurring with ADHD and generalized anxiety disorder (Cengher et al., 2021), while questionnaire studies show higher social‑anxiety and sensory‑avoidance scores in SM + ASD than SM‑only groups. They found that children with both SM and autism exhibited higher levels of social anxiety and sensory avoidance than those with SM alone (Ludlow et al., 2023; McKenna et al., 2017). Large neurodevelopmental cohorts add genetic weight: SM is over‑represented in Cornelia‑de‑Lange, Fragile X and Dup 7q11.23 syndromes, conditions already linked to autism (Nelson et al., 2016; Klein‑Tasman & Mervis, 2018). Family‑aggregation data echo this: 43% had a family history of autism (Sharkey & McNicholas, 2012).

### Gender

Most epidemiological and clinic‑based studies indicate a female preponderance in SM. In a systematic review, Kawai et al. (2020) estimated boy‑to‑girl ratios ranging from 1:0.4 to 1:2.6. Large clinic samples report similar patterns, for example, Steffenburg et al. (2018) found a ratio of 1:2.7 in Sweden, and Boneff‑Peng et al. (2023) reported 1:2.0 in China. Community studies from Finland, Norway and the Netherlands yield ratios between 1:1.5 and 1:2.1 (Kristensen, 2001; Kumpulainen et al., 1998; Muris et al., 2016).

By contrast, three school‑based surveys from Turkey and Bahrain observed equal or slightly higher male prevalence (Bergman et al., 2002; Karakaya et al., 2008; Marhoon et al., 2002). Methodological heterogeneity, including screening tools, age band and cultural norms, likely contribute to these discrepancies.

### Objectives of the Present Study

The present population-based study addresses these questions by linking individual level diagnostic records from the Norwegian Patient Register (2008–2023). By quantifying patterns of co-occurrence in an unselected national cohort, we investigate whether the current exclusion of autism from selective mutism is empirically justified and discuss the implications for clinical assessment and early intervention. In addition to autism, we examine several other frequently co-occurring conditions to illustrate the complexity of selective mutism presentations. We address the following two research questions (1) How common is autism among individuals who nevertheless receive an SM diagnosis in health services? and (2) Do age at diagnosis and gender distribution differ between SM cases with and without autism?

## Method

### Design and Participants

We identified a sample of 1,682 individuals aged 3–18 years residing in Norway who, between January 1, 2008, and April 30, 2023, had at least one documented diagnosis of selective mutism (SM; ICD-10 code F94.0). These diagnoses were recorded within the service areas of Mental Health Care for Children and Adolescents, Contracted Specialists in Mental Health Care, and Specialized Interdisciplinary Substance Abuse Treatment, as reported to the Norwegian Patient Registry (NPR). During the study period, all diagnoses recorded in the Norwegian Patient Registry (NPR) followed national coding guidance based on ICD-10 criteria (Helsen.no, 2024). In Norway, autism spectrum disorder and selective mutism are diagnosed exclusively within specialist health services (child and adolescent mental health services and pediatric habilitation). Diagnoses are assigned by board-certified specialists such as child and adolescent psychiatrists and clinical psychologists working in multidisciplinary teams and applying ICD-10 criteria, with DSM-5 cross-references where relevant. Primary care providers and schools do not issue these diagnoses. Consequently, while the data reflect clinician judgment, the diagnostic procedures are highly standardized and comparable to those used in many other countries.

Age was determined based on the date of diagnosis registration rather than the date of data extraction. As a result, individuals initially diagnosed with SM at ages 6–9 may appear in the 10–13 age group if they later received an autism diagnosis.

### Data Source

NPR data used for research purposes are fully anonymized and do not require ethical approval in Norway. According to Helsedata.no, the Norwegian Patient Registry (NPR) is a national health registry established by the Norwegian Directorate of Health in 1997 and operational since 2008. It systematically collects individual-level diagnostic data reported by both public and private specialized healthcare providers contracted by regional health authorities.

Identification numbers in NPR are encrypted to ensure strict confidentiality (Surén et al., 2012). Reporting to NPR is mandatory for specialized healthcare providers and is directly linked to the national reimbursement system. Diagnoses are recorded using ICD-10 codes, and access to NPR data is regulated under the Health Registry Act (Bakken et al., 2020).

## Measures

### Selective Mutism

Presence of an ICD-10 F94.0 code recorded in the Norwegian Patient Registry (NPR). Only registrations made by certified child psychiatrists or clinical psychologists in specialist care were included.

### Autism

Any ICD-10 F84.x code (F84.0–F84.9). Co-occurrence was coded in variables (ADHD F90.0, SAD F40.1, GAD F41.1, Tourette’s F95.2, DLD (F80) and MDD (F33).

### Gender

NPR supplies juridical sex (M/F). The NPR only records gender (boy/girl). We recognize that gender is a spectrum (Clark et al., 2021), but such data is not available in the registry.

### Age at First SM Registration

Calculated in years and grouped into 3–5, 6–9, 10–13, and 14–18 years to facilitate aggregated analysis.

### Cell Suppression

In line with NPR policy, any table cell containing fewer than five individuals is reported as “< 5”; the value 1 is used as a graphical placeholder to preserve scaling.

### Procedures

Anonymized, individual level data was obtained through formal application and approval procedures with NPR. 1 registration of SM (F94.0) between 1 January 2008 and 30 April 2023. Age 3–18 years at first SM registration. Registered residence in Norway at time of diagnosis.

The reliability of the dataset was supported by the mandatory reporting requirements for specialized healthcare providers, ensuring consistent application of ICD-10 criteria.

### Data Quality and Validity

The internal validity of the study was supported through the use of standardized diagnostic frameworks employed by specialists such as child psychiatrists and clinical psychologists. However, external validity may be limited, as findings are based exclusively on NPR data and may not generalize to populations outside of Norway.

Co-occurring diagnoses were analyzed, with special attention given to individuals with three or more concurrent conditions, acknowledging the potential impact of diagnostic overshadowing. Given the ICD-10 exclusion of autism from SM, the time interval between SM and subsequent autism diagnoses was analyzed to explore potential diagnostic delays. All diagnoses were made by certified specialists in child and adolescent psychiatry or clinical psychology.

### Statistical Analysis

Descriptive statistics were used to analyze gender distribution in the SM sample, age at first diagnosis for SM and autism. Microsoft Excel 365 was used for data organization and visualization purposes, including the generation of bar charts to illustrate patterns in co-occurrence and diagnosis timing.

Counts of SM cases were also cross-tabulated by female vs. male and ASD diagnosis (yes vs. no). A 2 × 2 chi-square test of independence was run in JASP (Version 0.19.3; JASP Team, 2023). To evaluate whether autism co-occurrence differed by gender. Effect size was reported as Cramér’s φ, with α = 0.05; all expected cell frequencies were ≥ 5.

Counts of children with SM were cross-tabulated by four age groups (3–5, 6–9, 10–13, 14–18 years) and presence/absence of autism. A chi square test of independence was run in JASP to evaluate whether autism co-occurrence varied by age; all expected cell frequencies exceeded 5. Effect size was expressed as Cramér’s V, and *p* <.05 (two-tailed) was considered statistically significant.

Statistical analyses also provide insights into the relationships between gender, age, and diagnostic patterns.

## Results

### Gender Distribution

**Fig. 1:**
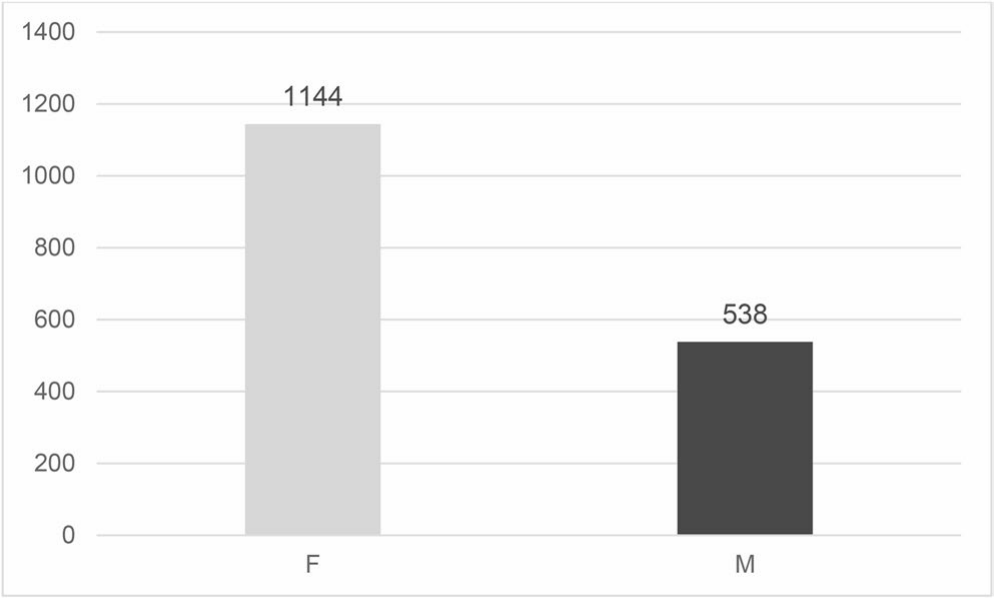
Gender distribution in the selective mutism sample. Data from NPR (2008–2023)

**Fig. 2:**
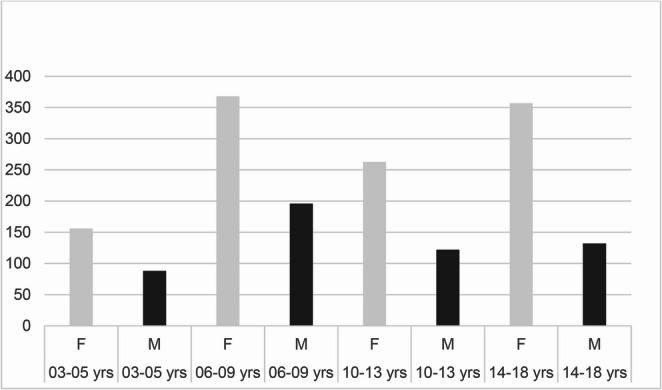
Selective mutism and gender distribution with data from NPR (2008–2023)

Across the full SM cohort (*N* = 1,682) girls outnumbered boys by roughly two to one (ratio 2.13:1). Case counts peaked in the 6–9-year group and remained high through adolescence (Figs. 1 and 2).

### Autism Co-Occurrence

Overall, 11.7% of children with SM also had a co-occurring autism diagnosis. A 4 × 2 chi-square test revealed a significant association between age group and autism diagnosis, χ²(3, *N* = 1,682) = 108.20, *p* <.001, Cramér’s V = 0.25, 95% CI (0.20, 0.30). This suggests that the likelihood of receiving an autism diagnosis increases with age among children with selective mutism.

**Fig. 3:**
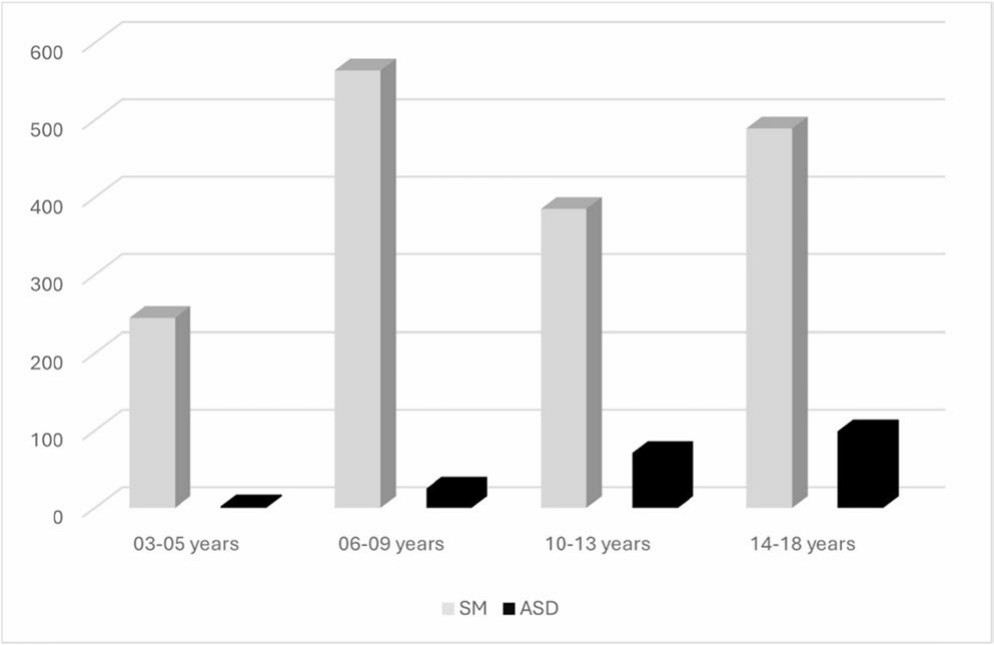
Selective mutism with co-occurring autism in this SM sample with data from NPR (2008–2023)

The data (Fig. 3) indicate that autism is increasingly recognized in later childhood, consistent with diagnostic delay and masking effects.

### Gender Differences

A separate 2 × 2 χ² test revealed that autism co-occurrence was significantly more common in boys (16.0%) than in girls (9.7%), χ²(1, *N* = 1,682) = 13.97, *p* <.001, φ = 0.09. Despite fewer boys overall, a larger proportion met autism criteria (M = 16% vs. F = 9.7%).

The percentage of girls and boys within the SM sample who also have each co-occuring diagnosis, calculated relative to the total number of girls and boys with SM, respectively. (*N* = 1,682, 68% were girls and 32% were boys).

### Additional Co-Occurrence

**Fig. 4:**
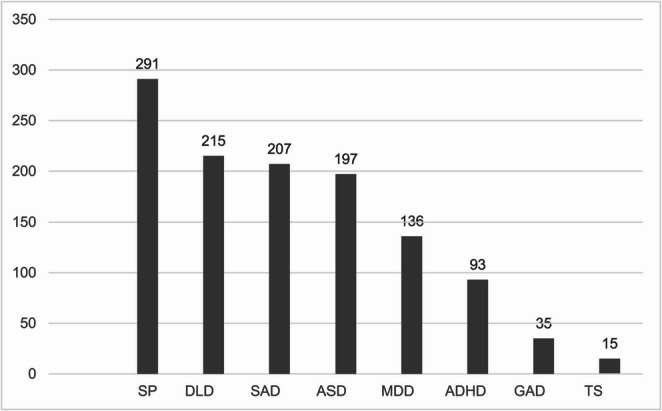
Co-occurring SM with data from NPR (2008–2023). Figure abbreviations: Social Phobia (SP), Developmental Language Disorder (DLD), Social Anxiety Disorder (SAD), Autism Spectrum Disorder (ASD), Major Depressive Disorder (MDD), Generalized Anxiety Disorder (GAD), Tourette Syndrome (TS)

Figure 4 summarizes other frequent co-occurring psychiatric and neuro-developmental diagnoses, underscoring the clinical complexity of the SM population.

Among the 1,682 Norwegian children diagnosed with selective mutism, co-occurrence is the rule rather than the exception. Social phobia, developmental language disorder, social anxiety disorder and autism spectrum disorder form the four largest clusters, together accounting for roughly half of the sample. Neurodevelopmental and anxiety-related conditions clearly dominate, underscoring the need for multidisciplinary assessment. Notably, 136 children, about 8% of the sample also received a major depressive disorder (MDD) diagnosis, showing that mood problems are a meaningful, if less common, part of the SM co-occurrence profile.

## Discussion

### Diagnostic Implications

The findings challenge the exclusion of autism as a co-occurring diagnosis for selective mutism, or vice versa, in ICD-10 and ICD-11. Despite the categorical distinction, this study identified a 11.7% co-occurrence of selective mutism and autism, indicating that these conditions frequently overlap in this clinical population. The delayed autism diagnoses observed in older age groups suggest that SM may initially mask autistic traits, leading to under-recognition and diagnostic overshadowing. This aligns with previous studies highlighting the limitations of rigid diagnostic boundaries in capturing the complexity of neurodevelopmental conditions (Muris & Ollendick, 2021; Steffenburg et al., 2018).

The exclusion criterion in ICD-10/11 may contribute to misclassification and delays in providing suitable help and intervention. Children diagnosed with SM at an early age, but later identified as autistic, may not receive appropriate support during critical developmental windows. Given the evidence of masking and freeze responses in both SM and autism (Vogel et al., 2022), distinguishing between these conditions requires a more nuanced diagnostic framework.

Sensory sensitivities may play a critical role in both SM and autism. Although the NPR dataset contains no direct sensory variables, converging evidence supports systematic sensory screening in SM assessments. Children with SM-only display reduced facial fixations under social-performance threat (Vogel et al., 2022), whereas autistic shutdowns are predominantly evoked by auditory, visual or tactile overload (Belek, 2018). Crucially, SM + ASD cases score about 0.6 standard deviations higher on Sensory-Avoidance than SM-only peers (Ludlow et al., 2023). A brief tool such as the Short Sensory Profile (Dunn, 1999) can therefore help clinicians decide whether a child’s silence is primarily anxiety-driven or mediated by sensory sensitivities. More research is needed to clarify whether such sensitivities are specific to SM or largely explained by comorbidity with autism and related neurodevelopmental conditions.

Selective mutism is classified as an anxiety disorder in DSM-5-TR and ICD-11 (APA, 2022; WHO, 2019). When viewed through this lens, anxiety-driven selective mutism may be mistaken for neurodevelopmental conditions because of phenotypic overlap, particularly in cases where undiagnosed autism is present (Muris & Ollendick, 2015, 2021). Our patient data illustrate such overlap and highlight a methodological challenge for research: without careful differential assessment, samples may inadvertently combine “pure” selective mutism with cases that also meet criteria, or show traits, for autism. In addition, language disorders are themselves classified as neurodevelopmental disorders (APA, 2022; WHO, 2019). This debate reflects broader discussions in the field, where some emphasize the anxiety basis of selective mutism, while others point to clinically meaningful intersections with autism profiles (Muris & Ollendick, 2021).

### Clinical Implications

In addition to the overlap between SM and autism, these data (Fig. 3) indicate that autism is increasingly recognized in later childhood. These figures echo the 63% autism rate and 4–6-year delay reported by Steffenburg et al. (2018) and reinforce concerns about diagnostic overshadowing.

In addition to the overlap between SM and autism, these data (Fig. 3) suggest that autism is often recognized later in childhood and they can co-occur. The numbers are in line with a Norwegian study reporting 7% Asperger (Kristensen & Torgersen, 2001) and with the 63% autism rate and diagnostic delay found by Steffenburg et al. (2018). The 63% figures might reflect referral and selection bias from a specialized neurodevelopmental center and therefore represent an enriched clinical sample, not a population estimate. Still these different numbers reinforce concerns about diagnostic overshadowing.

From an intervention perspective, traditional social anxiety treatments may not be suitable for children with both SM and ASD. For instance, forced exposure to social situations or behavioral reinforcement strategies commonly used for SM may inadvertently reinforce masking behaviors in autistic children (Pearson & Rose, 2021). Suzuki et al. (2020) emphasizes the importance of considering SM and ASD as separate conditions, requiring individualized intervention strategies. For instance, if SM is addressed without recognizing underlying autism, social exercises may backfire. Practicing eye contact may encourage masking rather than genuine social engagement (Pearson & Rose, 2021).

Because ICD-11 still lists autism as an exclusion for SM, many autistic children risk missing early, autism-specific understanding, as children with SM may initially be perceived solely within an anxiety framework, rather than as part of a broader neurodevelopmental profile.

### Gender

Across 11 published studies, eight report a female preponderance in SM. We found the Norwegian ratio to be 2.13 girls for every boy (M/F 1:2.13). Thus, our nationwide ratio lies within earlier reported ranges from other countries. Cultural context and referral bias may explain the few studies that find equal or male-biased ratios. It is also worth noting that not all these studies intend to examine gender distribution as their main goal, and they may vary due to method or cultural context. It should also be noted that our Norwegian figure is based on a relatively large sample in this context of 1,682 individuals.

Statistics Norway (2024) collects statistics in Norway and provide a valuable reference for assessing the general population’s gender distribution. This is relevant when comparing gender distribution among those with SM in this study. While caution is needed when generalizing findings from specific groups like SM, SSB data indicates no higher proportion of girls in the general population during the same period. Therefore, it appears there is a significant tendency for more girls to be diagnosed with SM compared to boys in Norway.

Despite fewer boys overall, a larger proportion met autism criteria (M = 16% vs. F = 9.7%).

Autism is diagnosed more often in boys than girls (≈ 3–4:1), though newer evidence suggests under-identification in girls may narrow this gap (Loomes et al., 2017; Maenner et al., 2023; Rødgaard et al., 2021b). In our sample (Table 1), boys with SM were more likely to have co-occurring autism, developmental language disorder, and tic disorders. Whereas girls with SM more often presented with anxiety and mood disorders (e.g., social phobia, GAD, MDD). ADHD rates were comparable across sexes.

**Table 1:** Selective mutism and co-occurring diagnoses distributed by gender. Data from NPR (January 1, 2008, to April 30, 2023)Selective mutism and co-occurring diagnoses distributed by gender. Data from NPR (January 1, 2008, to April 30, 2023)

Gender (SM)	ASD (%)	DLD (%)	TS (%)	ADHD (%)	SAD (%)	MDD (%)	SP (%)	GAD (%)
Female (*n* = 1,144)	9.70	10.84	0.17	5.60	12.93	10.05	18.71	2.88
Male (*n* = 538)	15.99	16.91	2.42	5.39	10.97	3.90	14.32	0.37

*ASD* Autism Spectrum Disorder, *DLD* Developmental Language Disorder, *TS* Tourette Syndrome, *ADHD* Attention-Deficit/Hyperactivity Disorder, *SAD* Social Anxiety Disorder, *MDD* Major Depressive Disorder, *SP* Social Phobia, *GAD* Generalized Anxiety Disorder

### Limitations

This study has some limitations that may affect the generalizability and interpretation of the findings. The data from the Norwegian Patient Register, only contains information on patients who have been in contact with the specialist healthcare services and as reporting errors are rare, but they can happen. This may mean that individuals with selective mutism who have not sought help through specialist care are not represented in the study. The study only includes a specific age group (3–18 years), which limits the ability to draw conclusions about adults with SM. It may be a certain time delay in the diagnosis process for co-occurring conditions which can affect the accuracy of the co-occurrence-analysis.

The diagnoses are based on ICD-10, which has stricter criteria for diagnosing SM compared to other systems, such as DSM-5, which may have led to underreporting of cooccurring diagnoses, such as autism. While NPR provides comprehensive national data, diagnostic entries depend on clinicians’ reporting practices, which may introduce misclassification, omission, or inconsistencies in coding (Helsedata.no, 2024). Variability in how clinicians interpret ICD-10 criteria can lead to differences in diagnostic thresholds, particularly in borderline cases of SM and autism. As autism is listed as an exclusion criterion for SM under ICD-10, individuals who receive e.g. an SM-diagnosis will not be seen as eligible to receive an autism-diagnosis, or vice versa. This diagnostic delay complicates interpretations of co-occurrence rates, as autism may be recorded years after SM rather than concurrently, with the current exclusion criterion present in the ICD-10 and ICD-11.

Additionally, due to the scale of NPR, technical issues such as missing records from system failures or data migration processes cannot be entirely ruled out.

Further, possible explanations for the observed co-occurrence may include true clinical overlap between selective mutism and autism, shared risk factors (e.g., anxiety profiles or genetic predispositions), and heightened clinical awareness leading to more frequent dual diagnoses.

Anonymization (* for 1–4 individuals) results in the loss of precise information in certain cells, which can complicate statistical testing. Aggregated data also prevents more advanced individual-level analyses and makes it impossible to determine how many children have multiple co-occurrence diagnoses simultaneously (e.g., SM + ADHD + ASD).

Gender and cultural differences can influence how selective mutism is diagnosed and understood. For instance, traits in boys may be overlooked or interpreted differently than in girls, creating biases in gender distribution. Different cultures may have varying perceptions of quiet behavior, which can affect how SM is reported.

### Research Directions

Future research should further investigate the interplay between SM and autism, particularly in the context of diagnostic overshadowing and late identification. Longitudinal studies examining developmental trajectories of children initially diagnosed with SM can provide deeper insights into whether SM is a precursor, a distinct condition, or a parallel manifestation of underlying autistic traits.

Although data from this study are based on diagnostic procedures that are highly standardized and comparable to diagnostic processes in other countries, future research could strengthen these findings by employing prospective designs with a higher control of usage of gold-standard diagnostic instruments (e.g., ADOS-2, ADI-R) and larger, population-based cohorts to more precisely establish rates of co-occurrence and clarify underlying mechanisms. Albeit abovementioned diagnostic instruments are the norm in diagnostic assessment in Norway, geographical differences occur, and this information was, for this study, not available.

Neuro-genetic studies should test the hypothesized shared pathways suggested by CNTNAP2 and 7q11.23 data. Previous research has identified potential genetic overlaps between these conditions (Nelson et al., 2016; Stein et al., 2011), but more work is needed to clarify how neurodevelopmental pathways contribute to selective mutism.

Finally, qualitative research focusing on lived experiences of individuals with SM and ASD could enhance clinical understanding.

## Conclusion

Using nation-wide registry data, we show that 11.7% of Norwegian children with selective mutism also meet autism criteria. As seen in our data, social anxiety, developmental language disorder and autism spectrum disorder constitute the four most frequent comorbidities in our cohort, together affecting almost half of all children with selective mutism. This dominance of neurodevelopmental and anxiety-related conditions highlights and challenges ICD-10/11’s exclusion rule and underscores the urgency of dual screening and tailored intervention that address both anxiety and neurodevelopmental needs.

It is important to have knowledge about SM, because starting intervention early leads to better outcomes (Rozenek et al., 2020), and the knowledge of causes, because like Cengher et al. (2021) indicates; for children with both selective mutism and autism it can be more challenging to find efficient and effective intervention options. Early dual screening is therefore crucial to deliver timely, tailored support and understanding.

## Data Availability

Data from the Norwegian Patient Registry analyzed in this study are not publicly available due to ethical restrictions.
